# Less Timely Initiation of Glucose-Lowering Medication Among Younger and Male Patients With Diabetes and Similar Initiation of Blood Pressure-Lowering Medication Across Age and Sex: Trends Between 2015 and 2020

**DOI:** 10.3389/fphar.2022.883103

**Published:** 2022-05-12

**Authors:** Martina Ambrož, Sieta T. de Vries, Klaas Hoogenberg, Petra Denig

**Affiliations:** ^1^ Department of Clinical Pharmacy and Pharmacology, University Medical Center Groningen, University of Groningen, Groningen, Netherlands; ^2^ Department of Internal Medicine, Martini Hospital, Groningen, Netherlands

**Keywords:** type 2 diabetes, glycosylated hemoglobin A1c (HbA1c), systolic blood pressure (SBP), age, sex differences, medication initiation

## Abstract

**Aims:** We aimed to assess trends in glycosylated hemoglobin A1c (HbA1c) and systolic blood pressure (SBP) thresholds at initiation of glucose- and blood pressure-lowering medication among patients with type 2 diabetes and assess the influence of age and sex on these trends.

**Materials and Methods:** We used the Groningen Initiative to ANalyze Type 2 diabetes Treatment (GIANTT) primary care database. Patients initiating a first non-insulin glucose-lowering or any blood pressure-lowering medication between 2015 and 2020 with an HbA1c or SBP measurement in the 120 days before initiation were included. We used multilevel regression analyses adjusted for potential confounders to assess the influence of calendar year, age or sex, and the interaction between calendar year and age or sex on trends in HbA1c and SBP thresholds at initiation of medication.

**Results:** We included 2,671 and 2,128 patients in the analyses of HbA1c and SBP thresholds, respectively. The overall mean HbA1c threshold at initiation of glucose-lowering medication significantly increased from 7.4% in 2015 to 8.0% in 2020 (*p* < 0.001), and particularly in the younger age groups. Compared to patients ≥80 years, patients aged 60–69 years initiated medication at lower levels mainly in the early years. Patients <60 years and between 70–79 years initiated medication at similar levels as patients ≥80 years. Females initiated medication at lower levels than males throughout the study period (*p* < 0.001). The mean SBP threshold at initiation of blood pressure-lowering medication varied from 145 to 149 mmHg without a clear trend (*p* = 0.676). There were no differences in SBP thresholds between patients of different ages or sex.

**Conclusion:** The rising trend in the HbA1c threshold for initiating glucose-lowering medication in the lower age groups was unexpected and requires further investigation. Males appear to receive less timely initiation of glucose-lowering medication than females. The lack of higher thresholds for the oldest age group or lower thresholds for the youngest age group in recent years is not in line with the age-related recommendations for personalized diabetes care and calls for health systems interventions.

## Introduction

Adequate treatment of risk factors, including glycosylated hemoglobin A1c (HbA1c) and systolic blood pressure (SBP), is important for people with type 2 diabetes mellitus (T2DM) to lower the risk of micro- and macrovascular complications (American Diabetes Association, 2021; Cosentino et al., 2019). When patients do not achieve recommended HbA1c and SBP target levels with lifestyle changes, medication treatment should be initiated. Timely initiation of medication treatment is important in order to achieve optimal targets and better outcomes (Martono et al., 2015; Martono et al., 2016; Khunti et al., 2019). Optimal targets, however, may differ between patients. In the last decade, treatment guidelines have incorporated more personalized recommendations based on patient factors such as age, frailty, cardiovascular risk, and patient preferences (Warnes et al., 2008; Nederlands Huisartsen Genootschap, 2011; Verenso, 2011; Mancia et al., 2013; Rydén et al., 2013; American Diabetes Assocation, 2015; Whelton et al., 2017; Federatie Medisch Specialisten, 2018; Nederlandse Internisten Vereniging, 2018; Williams et al., 2018; Cosentino et al., 2019; Nederlands Huisartsen Genootschap, 2019; American Diabetes Assocation, 2020; Barents et al., 2021). Older and more frail patients may require and prefer less aggressive treatment, given the shift in benefit-risk balance of tight risk factor control due to ageing (de Vries et al., 2015; Benetos et al., 2016; Sinclair et al., 2019; Ambrož et al., 2021a). Females with T2DM, on the other hand, may need more intensive treatment given their higher relative risks of cardiovascular and renal disease (Peters et al., 2014a; Peters et al., 2014b; Shen et al., 2017). Currently, it is unknown to what extent have these changes in treatment recommendations been applied in clinical practice. It is known that dissemination of new recommendations may need additional interventions targeting clinicians to be effective at changing practice patterns (Cliff et al., 2021).

To facilitate the effective and safe use of medication treatment, it is relevant to study drug utilization trends in the whole population as well as among specific subpopulations. A study looking at trends among Dutch T2DM patients showed that mean HbA1c and blood pressure levels decreased between 1998 and 2008 and were similar for different age categories (van Hateren et al., 2012). Another study showed a slight decrease in the proportion of patients treated with glucose-lowering medication between 1998 and 2013, with no significant sex differences in treatment or achieving targets in the later years (Hendriks et al., 2016). These studies also showed that treatment with blood pressure-lowering medication increased particularly in the early years, with only small differences between the sexes and age groups. Focusing on the initiation of medication, we previously observed little change in the mean HbA1c threshold at initiation of glucose-lowering medication between the years 2008–2014. Despite the changed recommendations towards more personalized treatment, we did not observe higher thresholds among older or frail patients over the years (Ambrož et al., 2021b). Furthermore, we observed that SBP thresholds at initiation of blood pressure-lowering treatment among T2DM patients decreased in the period 2009–2014, regardless of age or frailty (Ambrož et al., 2020).

Little is known about these treatment trends in the recent years. Our aim was to 1) assess trends in HbA1c and SBP thresholds at initiation of glucose- and of blood pressure-lowering treatment between the years 2015 and 2020, and 2) assess the influence of patients’ age and sex on these trends.

## Methods

### Study Design and Population

We conducted a repeated cross-sectional dynamic cohort study of the years 2015–2020. We used the Groningen Initiative to ANalyze Type 2 diabetes Treatment (GIANTT; www.giantt.nl) database, which contains anonymous electronic medical records data from T2DM patients treated in primary care in the north part of Netherlands. In Netherlands, the majority of T2DM patients are managed in primary care, often receiving 3-monthly check-ups by a nurse practitioner and yearly check-ups by their general practitioner. The methods used in this study were similar to those used in previous trend studies using the same database (Ambrož et al., 2020; Ambrož et al., 2021b).

Patients were included in the calendar year if they initiated treatment with a glucose- or blood pressure-lowering medication, had an HbA1c or SBP measurement, respectively, within 120 days before medication initiation and had at least 1 year of medical history in the GIANTT database. Medication initiation was defined as a prescription for a non-insulin glucose-lowering medication [anatomic therapeutic chemical (ATC) classification codes A10B] or any blood pressure-lowering medication (ATC codes C03, C04, C07, C08, C09) without a known prescription for any glucose- or blood pressure-lowering medication, respectively, in the preceding 365 days. We excluded patients who were diagnosed with diabetes before the age of 35 years because of the possibility that these were type 1 diabetes patients (Nielen et al., 2020). Patients who initiated treatment with three or more different glucose- or blood pressure-lowering medications, propranolol, or a loop diuretic were also excluded, since these treatments were more likely intensifying pre-existing treatment or were prescribed for other indications (Ambrož et al., 2020). Finally, all patients who had T2DM for more than 10 years were excluded from the analysis of HbA1c thresholds since it is unlikely that those were true initiators. We obtained an exemption letter from the University Medical Center Groningen Medical Ethics Review Board (reference number M19.235285) since in the Netherlands no approval is needed for studies using anonymous medical records.

### Outcomes and Explanatory Variables

Our two outcomes were the patients’ most recent HbA1c or SBP level in the 120 days before or on the day of glucose- or blood pressure-lowering medication initiation, respectively.

We included the following explanatory variables: calendar year of medication initiation, patients’ age or sex and the interaction between calendar year and age or sex. Age was calculated on 1 January of the calendar year in which the patient initiated treatment and was categorized in four groups (<60 years, 60–69 years, 70–79 years, and ≥80 years old) based on the different cut-offs observed among guidelines (Warnes et al., 2008; Nederlands Huisartsen Genootschap, 2011; Verenso, 2011; Mancia et al., 2013; Rydén et al., 2013; American Diabetes Association, 2015; Whelton et al., 2017; Federatie Medisch Specialisten, 2018; Nederlandse Internisten Vereniging, 2018; Williams et al., 2018; Cosentino et al., 2019; Nederlands Huisartsen Genootschap, 2019; American Diabetes Assocation, 2020; Barents et al., 2021). Sex was used as entered in the database.

### Confounders

Variables that could be associated with age or sex of the patient, that might affect the decision to initiate glucose- or blood pressure-lowering medication, and that were available in the GIANTT database were included as potential confounders. In particular, female sex and longer diabetes duration are known to be associated with higher age and possibly associated with less aggressive treatment. Also a higher number of chronic medication and poor renal function, which may prevent the initiation of additional medication, are known to be associated with higher age. On the other hand, elevated cardiovascular risk factors, which differs between age and sex groups, can be associated with more aggressive treatment. For more aggressive initiation of antihypertensive treatment, also a history of cardiovascular disease and smoking are likely to be confounders. Therefore, the following variables were included: sex or age in the analysis of the effect of age or sex, respectively, diabetes duration (0–1 year, 2–3 years, 4–5 years, 6–7 years, 8–9 years, or ≥10 years), presence (yes/no) of dyslipidemia [defined as low density lipoproteins (LDL) ≥2.5 mmol/L], estimated glomerular filtration rate (eGFR; ≤60 ml/min/1.73 m^2^ or >60 ml/min/1.73 m^2^), presence of albuminuria (albumin creatinine ratio ≥30 mg/g or albumin in 24 h urine ≥300 mg), body mass index (BMI; <24.9 kg/m^2^, 25–29.9 kg/m^2^, or ≥30 kg/m^2^), lipid-lowering treatment (no treatment or ≥1 classes) and number of all other prescribed chronic medications at initiation (used as a continuous variable). Additionally, the analyses of HbA1c thresholds were adjusted for SBP level (<140 mmHg or ≥140 mmHg) and blood pressure-lowering treatment (no treatment, 1 class, 2 classes, or ≥3 classes) and the analyses of SBP thresholds for HbA1c level (<7% or ≥7%), history of cardiovascular events (presence yes/no of myocardial disease, heart failure, or stroke), number and type of glucose-lowering treatment (none, one oral, two oral, or three or more orals and/or insulin) and smoking. More details about definitions and calculations of these variables have been described previously (Ambrož et al., 2021b; Ambrož et al., 2020).

### Missing Data

No data for the explanatory variables were missing. Confounders which had less than 20% of missing values were imputed using multiple imputation by chained equation (MICE). Imputing variables with large amounts of missing data would be expected to end up with larger error terms. For albuminuria, where more than 20% of patients had a missing value, we assumed these patients did not have albuminuria, since conducting this test is less common in patients without suspected kidney problems.

### Analyses

The same analyses were used for the HbA1c and SBP thresholds. Patient characteristics were analyzed descriptively per calendar year, age, and sex group. We conducted multilevel regression analyses with a two-level random intercept model to account for patients being nested within general practices. First, using the empty model which includes only the outcome, we calculated the intraclass correlation coefficient to assess the variance that is attributed to general practices. Next, we added the potential confounders to assess the overall trend over the years. This model was also used to analyze the trends in each age and sex group separately, where after applying Bonferroni correction for multiple testing the significance levels were set at *p* < 0.0125 for age and *p* < 0.025 for sex. Last, to assess the effect of age and sex over time, we added age or sex and the interaction between year and age or sex to the model. All analyses were conducted in Stata V.14 (Stata Corp., College Station, Texas).

## Results

There were 2,671 and 2,128 patients who met our in- and exclusion criteria included in the analyses of HbA1c and SBP thresholds, respectively (Supplementary Figures S1, S2). The number of included general practices ranged from 72 in 2015, 78 in 2016–2018, 76 in 2019 to 59 in 2020. The variance explained by the general practices was 6.9% and 5.5%, respectively.

### Trends in Glycosylated Hemoglobin A1c Thresholds

The number of patients initiating glucose-lowering medication per year ranged from 348 to 551 (Supplementary Figure S1). Thirty-three percent of the included patients were younger than 60 years, 12% were 80 years old or older, 45% were females and 87% initiated treatment with metformin (Table 1). The patient characteristics over the years and per age and sex groups are shown in Supplementary Tables S1–S3. Complete data were available for 76% of the patients.

**TABLE 1:** Characteristics of patients included in the glycosylated hemoglobin A1c (HbA1c) threshold analyses (*N* = 2,671).

Females; N (%)	1,205 (45)
Age in years; N (%)	
<60	894 (33)
60–69	792 (30)
70–79	669 (25)
≥80	316 (12)
HbA1c at initiation in %; mean ± SD	7.7 ± 1.5
Fasting glucose; mean ± SD ^+^	9.1 ± 3.0
Diabetes duration; N (%)	
0–1 year	967 (36)
2–3 years	480 (18)
4–5 years	482 (18)
6–7 years	418 (16)
8–9 years	324 (12)
Systolic blood pressure ≥140 mmHg; N (%) ^¶^	1,055 (39)
Body mass index in kg/m^2^; N (%) ^§^	
<25	345 (13)
25–29.9	948 (35)
≥30	1,309 (49)
Dyslipidemia; N (%) ^¥^	1,454 (54)
Estimated glomerular filtration rate ≤60 ml/min/1.73 m^2^; N (%) ^ɸ^	461 (17)
Albuminuria; N (%) ^||^	33 (1)
Number of chronic medications at initiation; mean ± SD	4.1 ± 3.1
Blood pressure-lowering medication at initiation; N (%)	
No treatment	1,025 (38)
1 medication class	599 (22)
2 medication classes	559 (21)
3 or more medication classes	488 (18)
Treated with a lipid-lowering medication; N (%)	1,373 (51)
Initiated medication; N (%)	
Metformin	2,328 (87)
Sulfonylurea	178 (7)
α-glucosidase inhibitors	1 (0)
Dipeptidyl peptidase 4 (DDP-4) inhibitor	5 (0)
Glucagon-like peptide-1 (GLP-1) agonist	2 (0)
Sodium-glucose transport protein 2 (SGLT2) inhibitor	1 (0)
Metformin + another medication	151 (6)
Sulfonylurea + another medication	5 (0)

Missing values: ^+^ 366 (14%); ^¶^ 303 (11%); ^§^ 69 (3%); ^¥^ 387 (14%); ^ɸ^ 248 (9%); ^||^ 792 (30%).

**FIGURE 1:**
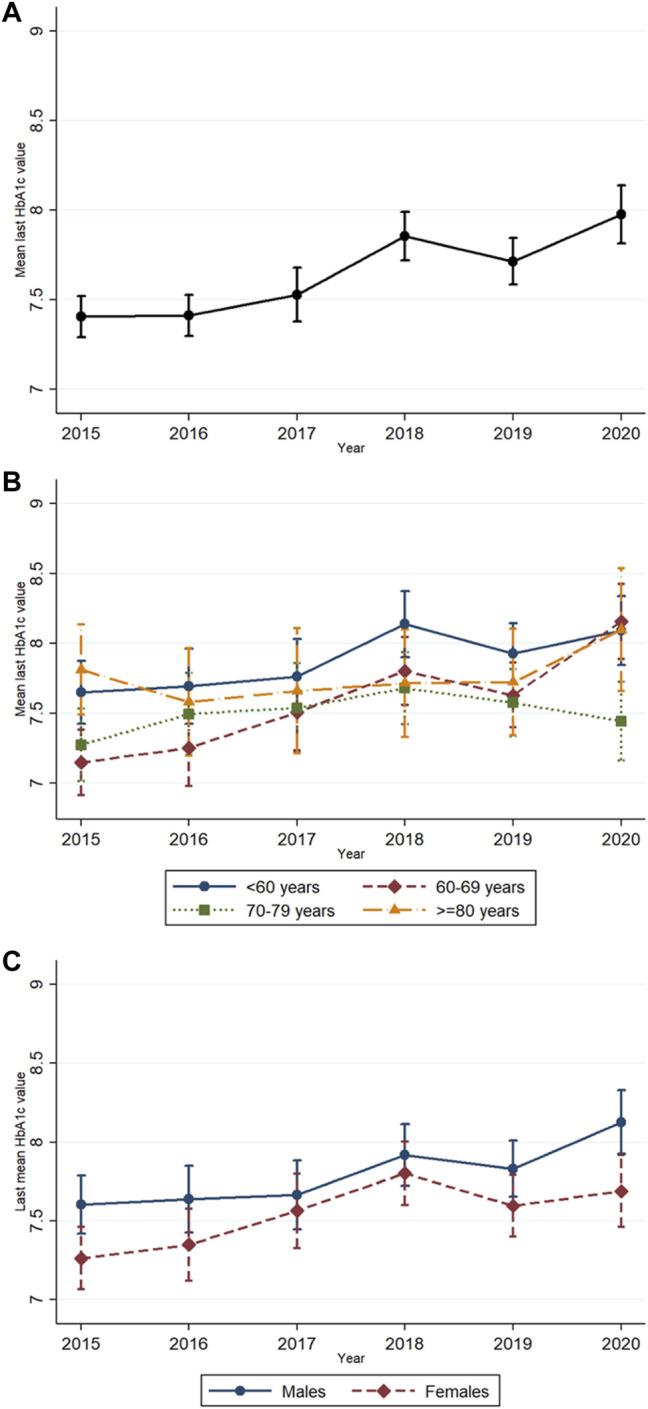
Mean last glycosylated hemoglobin A1c (HbA1c) levels adjusted for all potential confounders with 95% confidence intervals before/at initiation of glucose-lowering medication from 2015 to 2020 in **(A)** the whole population, **(B)** different age groups, and **(C)** by sex.

The overall mean HbA1c thresholds at initiation of glucose-lowering medication significantly increased over the years from 7.4% in 2015 to 8.0% in 2020 [linear trend, *β* (year) = 0.093, 95% CI 0.062, 0.124; *p* < 0.001; Figure 1A]. In the analysis per age group (Figure 1B), the mean HbA1c threshold significantly increased over time in patients younger than 60 years [linear trend, *β* (year) = 0.086, 95% CI 0.026, 0.147; *p* = 0.005] and those aged 60–69 years [linear trend, *β* (year) = 0.182, 95% CI 0.125, 0.239; *p* < 0.001]. No statistically significant linear nor quadratic trends were seen in the older age groups. In the analyses by sex (Figure 1C), the mean HbA1c threshold significantly increased over time in both males [linear trend, *β* (year) = 0.087, 95% CI 0.044, 0.130; *p* < 0.001] and females [linear trend, *β* (year) = 0.101, 95% CI 0.056, 0.146; *p* < 0.001].

Patients younger than 60 years initiated glucose-lowering treatment at somewhat higher HbA1c levels than older patients in most years (Figure 1B), but this age effect was not statistically significant (Table 2). On the other hand, patients aged 60–69 years initiated treatment at lower levels in the first years and at similar levels in the later years compared to patients aged 80 years or older (Table 2). Females initiated glucose-lowering treatment at significantly lower HbA1c thresholds than males, which was seen in all years (Figure 1C; Table 2).

**TABLE 2:** Influence of age and sex on glycosylated hemoglobin A1c thresholds.

	β	95% CI	*p*
Age			
Calendar year	0.036	−0.050, 0.122	0.412
Age <60 years	−0.092	−0.481, 0.297	0.642
Age 60–69 years	−0.692	−1.081, −0.303	**<0.001**
Age 70–79 years	−0.291	−0.688, 0.107	0.152
Age ≥80 years	Reference group		
Year * Age <60 years	0.056	−0.044, 0.156	0.273
Year * Age 60–69 years	0.141	0.039, 0.243	**0.007**
Year * Age 70–79 years	0.006	−0.099, 0.110	0.917
Year * Age ≥80 years	Reference group		
Sex			
Calendar year	0.093	0.062, 0.124	**<0.001**
Female	−0.252	−0.360, −0.144	**<0.001**
Male	Reference group		
Interaction female*year	Not significant		

The intraclass correlation coefficient (ICC) calculated from the empty model was 0.069.

Multilevel models were adjusted for diabetes duration, number of chronic medications at initiation, number of antihypertensive medication classes, systolic blood pressure, lipid-lowering medication, presence of albuminuria, presence of dyslipidemia, estimated glomerular filtration rate and body mass index, and sex or age in the age and sex analyses, respectively. Bold: significance at *p* < 0.0125 for age and *p* < 0.025 for sex.

### Trends in Systolic Blood Pressure Thresholds

The number of patients initiating blood pressure-lowering medication included in our analysis ranged from 272 to 419 (Supplementary Figure S2). Twenty-six percent of these patients were younger than 60 years, 17% were 80 years or older and 48% were females (Table 3). Patient characteristics over the years and by age and sex groups are shown in Supplementary Tables S4–S6. Complete data were available for 72% of the patients.

**TABLE 3:** Characteristics of included patients in the systolic blood pressure (BP) analyses (*N* = 2,128).

Females; N (%)	1,011 (48)
Age in years; N (%)	
<60	559 (26)
60–69	650 (31)
70–79	566 (27)
≥80	353 (17)
Systolic BP at initiation in mmHg; mean ± SD	146 ± 21
Diastolic BP at initiation in mmHg; mean ± SD^+^	82 ± 13
Diabetes duration; N (%)	
0–1 year	273 (13)
2–3 years	316 (15)
4–5 years	276 (13)
6–7 years	307 (14)
8–9 years	250 (12)
≥10 years	706 (33)
Glycated hemoglobin A1c <7%; N (%)^¶^	1,072 (50)
Body mass index in kg/m^2^; N (%)^§^	
<25	392 (18)
25–29.9	823 (39)
≥30	866 (41)
Dyslipidemia; N (%)^¥^	954 (45)
Estimated glomerular filtration rate ≤60 ml/min/1.73m^2^; N (%)^ɸ^	368 (17)
Albuminuria; N (%)^||^	68 (3)
Smoking; N (%)^!^	371 (17)
History of cardiovascular disease; N (%)	
Myocardial disease^a^	213 (10)
Heart failure^b^	84 (4)
Stroke^c^	114 (5)
Number of chronic medications at initiation; mean ± SD	3.8 ± 2.8
Glucose-lowering medication at initiation; N (%)	
No medication	760 (36)
1 oral	736 (35)
2 orals	318 (15)
3 orals or more and/or insulin	314 (15)
Treated with lipid-lowering medication; N (%)	1,073 (50)
Initiated medication; N (%)	
Renin-angiotensin-aldosterone system inhibitor	870 (41)
Combination of antihypertensives	445 (21)
Beta blocker	345 (16)
Diuretic	260 (12)
Calcium channel blocker	208 (10)

Missing values: ^+^ 3 (0%); ^¶^ 133 (6%); ^§^ 47 (2%); ^¥^ 379 (18%); ^ɸ^ 237 (11%); ^||^ 552 (26%); ^**!**^ 330 (16%).

a Acute myocardial infarction (International Classification of Primary Care [ICPC] code K75) in the last year or other/chronic ischemic heart disease (ICPC, code K76) anytime in history.

b Heart failure (ICPC, code K77) anytime in history.

c Transient cerebral ischemia (ICPC, code K89) in the last year or stroke/cerebrovascular incident (ICPC, code K90) anytime in history.

**FIGURE 2:**
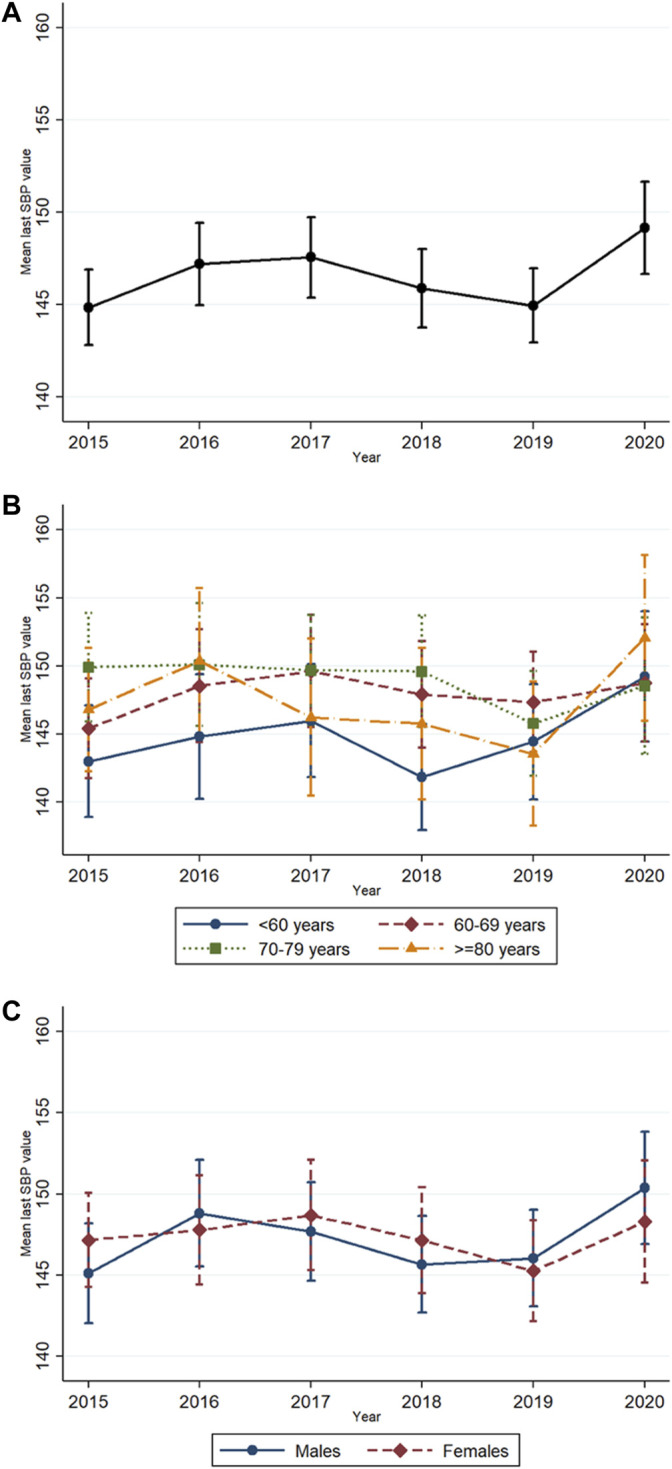
Mean last systolic blood pressure (SBP) levels adjusted for all potential confounders with 95% confidence intervals before/at initiation of blood pressure-lowering medication from 2015 to 2020 in **(A)** the whole population, **(B)** different age groups, and **(C)** by sex.

The mean SBP level at initiation of blood pressure-lowering medication rose from 145 mmHg in 2015 to 148 mmHg in 2017, dropped to 145 mmHg in 2019 and went up to 149 mmHg in 2020 (Figure 2A). This was not a significant linear or quadratic trend. There were also no statistically significant trends in the separate age and sex groups.

No significant differences in SBP levels at initiation of blood pressure-lowering medication based on age (Figure 2B) and sex (Figure 2C) were observed (Table 4). The interactions between age or sex and year were also not statistically significant (Table 4).

**TABLE 4:** Influence of age and sex on systolic blood pressure thresholds.

	β	95% CI	*p*
Age			
Calendar year	0.104	−0.426, −0.634	0.723
Age <60 years	−2.608	−5.909, 0.693	0.122
Age 60–69 years	0.523	−2.468, 3.513	0.732
Age 70–79 years	1.499	−1.248, 4.404	0.274
Age ≥80 years	Reference group		
Interactions with year	None are significant		
Sex			
Calendar year	0.113	−0.418, 0.644	0.676
Female	0.170	−1.589, 1.929	0.850
Male	Reference group		
Interaction female*year	Not significant		

The intraclass correlation coefficient (ICC) calculated from the empty model was 0.055.

Multilevel models were adjusted for diabetes duration, smoking status, number of chronic medications at initiation, number and/or type of glucose-lowering medication, lipid-lowering medication, presence of albuminuria, presence of dyslipidemia, hemoglobin A1C, history of cardiovascular events, estimated glomerular filtration rate, body mass index, and sex or age in the age and sex analyses, respectively.

## Discussion

This study shows that the HbA1c thresholds at initiation of glucose-lowering medication increased over the years 2015–2020. This increase was particularly seen in younger age groups and in both males and females. Females generally initiated medication at lower HbA1c thresholds than males. Patients aged 60–69 years initiated medication at lower levels in the first years and at similar levels in the later years compared to patients aged 80 years or older. Patients under 60 years and between 70–79 years initiated medication at similar levels as patients of 80 years and older. The SBP thresholds at initiation of blood pressure-lowering medication remained relatively stable over the study period regardless of age or sex.

We previously observed a rising trend in HbA1c thresholds for medication initiation between 2011 and 2014 (Ambrož et al., 2021b). The current study adds to this knowledge that this upward trend continued up to the year 2020. Furthermore, the decreasing trend observed in SBP thresholds between 2009 and 2014 (Ambrož et al., 2021b) appears to have stabilized after 2014. Looking at studies conducted in other countries, a mixed picture emerges. One study conducted in Denmark showed a decrease in mean pre-treatment HbA1c level between 2000 (9.2%) and 2011 (7.3%) followed by an increase to 7.9% in 2017 (Knudsen et al., 2020). These results suggest that prescribers in both Denmark and Netherlands became less strict regarding the initiation of glucose-lowering medication in recent years. In contrast, a study in the United Kingdom (UK) observed no changes in HbA1c level at initiation of medication from 2010 to 2017 (Dennis et al., 2019). The HbA1c threshold in 2017, however, was 8.6% in this UK study, which is much higher than the levels observed in Denmark or Netherlands. Differences in diabetes care between countries have been observed before and could be linked to organizational differences of healthcare systems (Si et al., 2010; Stone et al., 2013).

Most guidelines recommend initiating glucose-lowering medication at HbA1c levels above 6.5% or 7% in younger patients and above 8% or 8.5% in older patients (Verenso, 2011; Rydén et al., 2013; American Diabetes Association, 2015; Nederlandse Internisten Vereniging, 2018; American Diabetes Assocation, 2020; Barents et al., 2021). Surprisingly, we did not observe significant differentiation regarding the HbA1c levels at initiation of medication based on age. In particular, we observed similar HbA1c thresholds in patients under 60 years and those aged 80 years or older. These findings indicate potential undertreatment of hyperglycemia in younger patients, who initiated at mean HbA1c thresholds higher than 7.5% or even 8% in 2020. The recommended SBP threshold for initiation of blood pressure-lowering medication is 130 mmHg or 140 mmHg in younger patients and 150 mmHg or 160 mmHg in older patients (Mancia et al., 2013; Rydén et al., 2013; Whelton et al., 2017; Williams et al., 2018; Nederlands Huisartsen Genootschap, 2019). We observed mean SBP thresholds in all age groups ranging between 140 and 150 mmHg. These results indicate potential undertreatment of younger patients and potential overtreatment of older patients. Undertreatment of diabetes in younger or male patients has also been shown in Norway and Spain and could be caused by barriers at clinician, patient, and/or healthcare system level (Khunti et al., 2019; Andreozzi et al., 2020). Undertreatment of both hyperglycemia and hypertension in this population is of concern since this can lead to more complications (Holman et al., 2008a; Holman et al., 2008b; Hayward et al., 2015). Although therapeutic inertia in T2DM has been a well-known problem for many years, it seems that this lack of timely initiation did not change much over the last decade (Khunti et al., 2018). More emphasis and involvement of other healthcare professionals, such as pharmacists, might help reduce clinical inertia (Fazel et al., 2017; Meredith et al., 2021). On the other hand, overtreatment among older T2DM patients has received a lot of attention in the past decade (Oktora et al., 2021) and our study suggests that potential overtreatment at initiation of glucose-lowering medication has decreased over time. That was, however, not the case at initiation of blood pressure-lowering medication, which is of concern, since older patients are more vulnerable for hypotension-related adverse events (Ambrož et al., 2021a).

Throughout the study period, we observed higher HbA1c thresholds at initiation of glucose-lowering medication in males compared to females. A post-hoc analysis of sex differences in the years 2008–2014 showed the same differences (Supplementary Figure S3A). Differences in screening rates are an unlikely explanation, since a recent systematic review observed no clear sex differences in the assessment of cardiovascular risk factors, such as SBP and HbA1c levels, in type 2 diabetes patients (de Jong et al., 2021). However, it could partly be due to later diabetes diagnosis among males. A previous study showed higher HbA1c levels in males than females at diagnosis of diabetes (Wright et al., 2020). Earlier initiation of glucose-lowering medication in females could also be a consequence of their increased relative risk for cardiovascular and renal disease (Peters et al., 2014a; Peters et al., 2014b; Shen et al., 2017). Additionally, no sex differences in SBP thresholds were observed between 2015 and 2020, but between 2007 and 2009 females initiated blood pressure-lowering treatment at higher SBP thresholds than males (Supplementary Figure S3B). This could indicate that some previously identified sex- or gender-related issues leading to undertreatment of cardiovascular diseases among females are diminishing (Vogel et al., 2021).

An intriguing finding was that both mean HbA1c and mean SBP thresholds were highest in 2020. For the HbA1c threshold this could be a continuation of the rising trend over the years, but this is not the case for the SBP threshold. There are indications that the COVID-19 pandemic influenced diabetes care in the year 2020 in Netherlands (InEen, 2020). Other studies have shown decreases in screening rates, consultations and patient use of healthcare services due to fear of COVID-19 infection, as well as worse glycemic and blood pressure control after the beginning of COVID-19 pandemic (Endo et al., 2022). The impact of this could differ per age group, driven by differences in fear, comorbidity, and frailty. More evidence is needed to assess whether our observations in 2020 were due to the COVID-19 pandemic.

A strength of our study is the use of a large database of real world data from electronic medical records, representing a range of Dutch general practices from urban and rural areas. Data undergo data entry error checks before being imported in the GIANTT database, increasing the internal validity. Using medical record data also brings some limitations. First, this is a dynamic cohort and the variation between years could in part be due to a variation between the participating general practices. We conducted additional analyses of trends in HbA1c and SBP thresholds including only the 59 general practices that were present in the whole period up to the year 2020, which showed similar results (data not shown). Secondly, some patients may not be true initiators but people who moved to a general practice participating in GIANTT while already using medication. Although we included only patients with a medical history of 1 year, this may not prevent the inclusion of some patients that have been treated with glucose-lowering or blood pressure-lowering medication by other healthcare professionals in the past. We used an arbitrary cutoff of 10 years to exclude patients that were unlikely to be initiators. A previous study conducted in Netherlands showed that around 20% of patients have not yet started medication treatment 3 years after their diagnosis (Spoelstra et al., 2004). Since it is not known what the maximum time to initiation is in our study population, we conducted a post-hoc analysis of the HbA1c thresholds including only patients with a maximum duration of diabetes at medication initiation of 5 years, which showed similar results (data not shown). A post-hoc analysis of SBP thresholds excluding all patients with a history of cardiovascular disease also did not change the results (data not shown). Another limitation of using medical record data is that we had missing data for some potential confounders, but we used multiple imputation for confounders with less than 20% missing data to reduce possible bias. Additionally, we conducted a post-hoc analysis also imputing the albuminuria values with more than 20% missing data, which did not change the results (data not shown). Finally, the study was conducted in Dutch primary care and the results may be more generalizable to countries with similar diabetes care systems.

To conclude, the rising trend in the HbA1c threshold for initiating glucose-lowering medication in the lower age groups was unexpected and requires further investigation. The lack of lower thresholds for the youngest age group and higher thresholds for the oldest age group at initiation of glucose- and blood pressure-lowering drugs calls for interventions to support age-related personalized diabetes treatment. Multicomponent interventions targeting clinicians are seen as effective type of interventions (Harvey and Kitson, 2015; Cliff et al., 2021). This could include educational programs, implementation of decision support tools with specific alerts, feedback reports and other health system interventions. Our finding that males received less timely initiation of glucose-lowering medication than females also need attention. Studies are needed to explore the reasons for as well as the implications of this sex difference. The results of our trend study provide valuable insight into the translation of guideline recommendations into clinical practice, shows areas with room for improvement and can help policy makers to tailor future interventions enhancing appropriate treatment for all patients.

## Data Availability

The datasets used and/or analyzed during the current study are available on reasonable request and according to procedures as stipulated on www.giantt.nl. Requests to access these datasets should be directed to Petra Denig, p.denig@umcg.nl.

## References

[B1] Ambrož M. de Vries S. T. Hoogenberg K. Denig P. (2021). Older Age, Polypharmacy, and Low Systolic Blood Pressure Are Associated with More Hypotension-Related Adverse Events in Patients with Type 2 Diabetes Treated with Antihypertensives. Front. Pharmacol. 12, 728911. 10.3389/fphar.2021.728911 34630105 PMC8497792

[B2] Ambrož M. de Vries S. T. Hoogenberg K. Denig P. (2021). Trends in HbA1c Thresholds for Initiation of Hypoglycemic Agents: Impact of Changed Recommendations for Older and Frail Patients. Pharmacoepidemiol. Drug Saf. 30 (1), 37–44. 10.1002/pds.5129 32955156 PMC7756585

[B3] Ambrož M. de Vries S. T. Sidorenkov G. Hoogenberg K. Denig P. (2020). Changes in Blood Pressure Thresholds for Initiating Antihypertensive Medication in Patients with Diabetes: a Repeated Cross-Sectional Study Focusing on the Impact of Age and Frailty. BMJ Open 10 (9), e037694. 10.1136/bmjopen-2020-037694 PMC7485238 32912988

[B4] American Diabetes Association (ADA) (2021). 10. Cardiovascular Disease and Risk Management: Standards of Medical Care in Diabetes-2021. Diabetes Care 44 (Suppl. 1), S125–S50. 10.2337/dc21-S010 33298421

[B7] American Diabetes Association (ADA) (2015). (6) Glycemic Targets. Diabetes Care 38 Suppl (Suppl. l), S33–S40. 10.2337/dc15-S009 25537705

[B5] American Diabetes Assocation (ADA) (2020). 12. Older Adults: Standards of Medical Care in Diabetes-2020. Diabetes Care 43 (Suppl. 1), S152–S62. 10.2337/dc20-S012 31862755

[B6] Andreozzi F. Candido R. Corrao S. Fornengo R. Giancaterini A. Ponzani P. (2020). Clinical Inertia Is the Enemy of Therapeutic success in the Management of Diabetes and its Complications: a Narrative Literature Review. Diabetol. Metab. Syndr. 12, 52. 10.1186/s13098-020-00559-7 32565924 PMC7301473

[B8] Barents E. Bilo H. Bouma M. Dankers M. Rooij A. D. Hart H. (2021). NHG-Standaard Diabetes Mellitus Type 2 (Versie 5.5). Huisarts en Wetenschap. Utrecht: NHG. Available at: https://richtlijnen.nhg.org/ .

[B9] Barrot-de la Puente J. Mata-Cases M. Franch-Nadal J. Mundet-Tudurí X. Casellas A. Fernandez-Real J. M. (2015). Older Type 2 Diabetic Patients Are More Likely to Achieve Glycaemic and Cardiovascular Risk Factors Targets Than Younger Patients: Analysis of a Primary Care Database. Int. J. Clin. Pract. 69 (12), 1486–1495. 10.1111/ijcp.12741 26422335 PMC5054846

[B10] Benetos A. Bulpitt C. J. Petrovic M. Ungar A. Agabiti Rosei E. Cherubini A. (2016). An Expert Opinion from the European Society of Hypertension-European Union Geriatric Medicine Society Working Group on the Management of Hypertension in Very Old, Frail Subjects. Hypertension 67 (5), 820–825. 10.1161/HYPERTENSIONAHA.115.07020 26975708

[B11] Cliff B. Q. Avanceña A. L. V. Hirth R. A. Lee S. D. (2021). The Impact of Choosing Wisely Interventions on Low-Value Medical Services: A Systematic Review. Milbank Q. 99 (4), 1024–1058. 10.1111/1468-0009.12531 34402553 PMC8718584

[B12] Cosentino F. Grant P. J. Aboyans V. Bailey C. J. Ceriello A. Delgado V. (2019). 2019 ESC Guidelines on Diabetes, Pre-diabetes, and Cardiovascular Diseases Developed in Collaboration with the EASD. Eur. Heart J. 41 (2), 255–323. 10.1093/eurheartj/ehz486 31497854

[B13] de Jong M. Peters S. A. E. de Ritter R. van der Kallen C. J. H. Sep S. J. S. Woodward M. (2021). Sex Disparities in Cardiovascular Risk Factor Assessment and Screening for Diabetes-Related Complications in Individuals with Diabetes: A Systematic Review. Front. Endocrinol. 12, 617902. 10.3389/fendo.2021.617902 PMC8043152 33859615

[B14] de Vries S. T. de Vries F. M. Dekker T. Haaijer-Ruskamp F. M. de Zeeuw D. Ranchor A. V. (2015). The Role of Patients' Age on Their Preferences for Choosing Additional Blood Pressure-Lowering Drugs: A Discrete Choice Experiment in Patients with Diabetes. PLoS One 10 (10), e0139755. 10.1371/journal.pone.0139755 26445349 PMC4596700

[B15] Dennis J. M. Henley W. E. McGovern A. P. Farmer A. J. Sattar N. Holman R. R. (2019). Time Trends in Prescribing of Type 2 Diabetes Drugs, Glycaemic Response and Risk Factors: A Retrospective Analysis of Primary Care Data, 2010-2017. Diabetes Obes. Metab. 21 (7), 1576–1584. 10.1111/dom.13687 30828962 PMC6618851

[B16] Endo K. Miki T. Itoh T. Kubo H. Ito R. Ohno K. (2022). Impact of the COVID-19 Pandemic on Glycemic Control and Blood Pressure Control in Patients with Diabetes in Japan. Intern. Med. 61 (1), 37–48. 10.2169/internalmedicine.8041-21 34980759 PMC8810256

[B17] Fazel M. T. Bagalagel A. Lee J. K. Martin J. R. Slack M. K. (2017). Impact of Diabetes Care by Pharmacists as Part of Health Care Team in Ambulatory Settings: A Systematic Review and Meta-Analysis. Ann. Pharmacother. 51 (10), 890–907. 10.1177/1060028017711454 28573873

[B44] Federatie Medisch Specialisten (FMS) (2018). Addendum (Kwetsbare) Ouderen Bij CVRM [Addendum (Frail) Elderly and Cardiovascular Risk Management]. Federatie Medisch Specialisten.

[B20] Harvey G. Kitson A. (2015). Translating Evidence into Healthcare Policy and Practice: Single versus Multi-Faceted Implementation Strategies - Is There a Simple Answer to a Complex Question? Int. J. Health Pol. Manag 4 (3), 123–126. 10.15171/ijhpm.2015.54 PMC4357977 25774368

[B21] Hayward R. A. Reaven P. D. Emanuele N. V. Bahn G. D. Reda D. J. Ge L. (2015). Follow-up of Glycemic Control and Cardiovascular Outcomes in Type 2 Diabetes. N. Engl. J. Med. 373 (23), 978–206. 10.1056/NEJMc1508386 26332555

[B22] Hendriks S. H. van Hateren K. J. Groenier K. H. Houweling S. T. Maas A. H. Kleefstra N. (2016). Sex Differences in the Quality of Diabetes Care in the Netherlands (ZODIAC-45). PLOS ONE 10 (12), e0145907. 10.1371/journal.pone.0145907 PMC4703132 26713444

[B23] Holman R. R. Paul S. K. Bethel M. A. Matthews D. R. Neil H. A. (2008). 10-year Follow-Up of Intensive Glucose Control in Type 2 Diabetes. N. Engl. J. Med. 359 (15), 1577–1589. 10.1056/NEJMoa0806470 18784090

[B24] Holman R. R. Paul S. K. Bethel M. A. Neil H. A. Matthews D. R. (2008). Long-term Follow-Up after Tight Control of Blood Pressure in Type 2 Diabetes. N. Engl. J. Med. 359 (15), 1565–1576. 10.1056/NEJMoa0806359 18784091

[B25] InEen (2020). Rapportage zorggroepen diabetes mellitus, VRM, COPD en asthma [Transparent chronic care 2020: Report care groups 2020 diabetes mellitus, CVD, COPD and asthma]. InEen. Available at: https://ineen.nl/wp-content/uploads/2021/06/Benchmark-Transparante-Ketenzorg-2020.pdf .

[B26] Khunti K. Gomes M. B. Pocock S. Shestakova M. V. Pintat S. Fenici P. (2018). Therapeutic Inertia in the Treatment of Hyperglycaemia in Patients with Type 2 Diabetes: A Systematic Review. Diabetes Obes. Metab. 20 (2), 427–437. 10.1111/dom.13088 28834075 PMC5813232

[B27] Khunti S. Khunti K. Seidu S. (2019). Therapeutic Inertia in Type 2 Diabetes: Prevalence, Causes, Consequences and Methods to Overcome Inertia. Ther. Adv. Endocrinol. Metab. 10, 2042018819844694. 10.1177/2042018819844694 31105931 PMC6502982

[B28] Knudsen J. S. Hulman A. Rønn P. F. Lauritzen T. Sørensen H. T. Witte D. R. (2020). Trends in HbA1c and LDL Cholesterol in Patients with Type 2 Diabetes Receiving First-Time Treatment in Northern Denmark, 2000-2017: Population-Based Sequential Cross-Sectional Analysis. Diabetes Care 43 (2), e17–e9. 10.2337/dc19-0527 31796572

[B29] Laing S. Johnston S. (2021). Estimated Impact of COVID-19 on Preventive Care Service Delivery: an Observational Cohort Study. BMC Health Serv. Res. 21 (1), 1107. 10.1186/s12913-021-07131-7 34656114 PMC8520349

[B30] Lidin M. Lyngå P. Kinch-Westerdahl A. Nymark C. (2021). Patient Delay Prior to Care-Seeking in Acute Myocardial Infarction during the Outbreak of the Coronavirus SARS-CoV2 Pandemic. Eur. J. Cardiovasc. Nurs. 20 (8), 752–759. 10.1093/eurjcn/zvab087 34718511

[B31] Mancia G. Fagard R. Narkiewicz K. Redón J. Zanchetti A. Böhm M. (2013). 2013 ESH/ESC Guidelines for the Management of Arterial Hypertension: the Task Force for the Management of Arterial Hypertension of the European Society of Hypertension (ESH) and of the European Society of Cardiology (ESC). J. Hypertens. 31 (7), 1281–1357. 10.1097/01.hjh.0000431740.32696.cc 23817082

[B32] Martono D. P. Hak E. Lambers Heerspink H. Wilffert B. Denig P. (2016). Predictors of HbA1c Levels in Patients Initiating Metformin. Curr. Med. Res. Opin. 32 (12), 2021–2028. 10.1080/03007995.2016.1227774 27552675

[B33] Martono D. P. Lub R. Lambers Heerspink H. J. Hak E. Wilffert B. Denig P. (2015). Predictors of Response in Initial Users of Metformin and Sulphonylurea Derivatives: a Systematic Review. Diabet Med. 32 (7), 853–864. 10.1111/dme.12688 25582542

[B34] Meredith A. H. Buatois E. M. Krenz J. R. Walroth T. Shenk M. Triboletti J. S. (2021). Assessment of Clinical Inertia in People with Diabetes within Primary Care. J. Eval. Clin. Pract. 27 (2), 365–370. 10.1111/jep.13429 32548871

[B18] Nederlands Huisartsen Genootschap (NHG) (2011). Multidisciplinaire Richtlijn Cardiovasculair Risicomanagement [Multidisciplinary Guidelines Cardiovascular Risk Management]. Nederlands Huisartsen Genootschap.

[B35] Nielen M. Poos R. Korevaar J. (2020). Diabetes mellitus in Nederland. Prevalentie en incidentie: heden, verleden en toekomst. Utrecht: Nivel.

[B19] Nederlands Huisartsen Genootschap (NHG) (2019). NHG-standaard Cardiovasculair Risicomanagement [Multidisciplinary Guidelines Cardiovascular Risk Management]. Nederlands Huisartsen Genootschap.

[B49] Nederlandse Internisten Vereniging (NIV) (2018). Diabetes Mellitus Type 2 Bij Ouderen. Utrecht: Federatie Medisch Specialisten.

[B36] Oktora M. P. Kerr K. P. Hak E. Denig P. (2021). Rates, Determinants and success of Implementing Deprescribing in People with Type 2 Diabetes: A Scoping Review. Diabet Med. 38 (2), e14408. 10.1111/dme.14408 32969063 PMC7891362

[B37] Peters S. A. Huxley R. R. Woodward M. (2014). Diabetes as a Risk Factor for Stroke in Women Compared with Men: a Systematic Review and Meta-Analysis of 64 Cohorts, Including 775,385 Individuals and 12,539 Strokes. Lancet 383 (9933), 1973–1980. 10.1016/S0140-6736(14)60040-4 24613026

[B38] Peters S. A. Huxley R. R. Woodward M. (2014). Diabetes as Risk Factor for Incident Coronary Heart Disease in Women Compared with Men: a Systematic Review and Meta-Analysis of 64 Cohorts Including 858,507 Individuals and 28,203 Coronary Events. Diabetologia 57 (8), 1542–1551. 10.1007/s00125-014-3260-6 24859435

[B39] Rydén L. Rydén L. Grant P. J. Anker S. D. Berne C. Cosentino F. (2013). ESC Guidelines on Diabetes, Pre-diabetes, and Cardiovascular Diseases Developed in Collaboration with the EASD: the Task Force on Diabetes, Pre-diabetes, and Cardiovascular Diseases of the European Society of Cardiology (ESC) and Developed in Collaboration with the European Association for the Study of Diabetes (EASD). Eur. Heart J. 34 (39), 3035–3087. 10.1093/eurheartj/eht108 23996285

[B40] Seidu S. Hambling C. Holmes P. Fernando K. Campbell N. S. Davies S. (2022). The Impact of the COVID Pandemic on Primary Care Diabetes Services in the UK: A Cross-Sectional National Survey of Views of Health Professionals Delivering Diabetes Care. Prim. Care Diabetes S1751-9918 (21), 00231–X. 10.1016/j.pcd.2021.12.015 PMC8754561 35033477

[B41] Shen Y. Cai R. Sun J. Dong X. Huang R. Tian S. (2017). Diabetes Mellitus as a Risk Factor for Incident Chronic Kidney Disease and End-Stage Renal Disease in Women Compared with Men: a Systematic Review and Meta-Analysis. Endocrine 55 (1), 66–76. 10.1007/s12020-016-1014-6 27477292

[B42] Si D. Bailie R. Wang Z. Weeramanthri T. (2010). Comparison of Diabetes Management in Five Countries for General and Indigenous Populations: an Internet-Based Review. BMC Health Serv. Res. 10 (1), 169. 10.1186/1472-6963-10-169 20553622 PMC2903584

[B43] Sinclair A. J. Abdelhafiz A. H. Forbes A. Munshi M. (2019). Evidence-based Diabetes Care for Older People with Type 2 Diabetes: a Critical Review. Diabet Med. 36 (4), 399–413. 10.1111/dme.13859 30411402

[B45] Spoelstra J. A. Stolk R. P. Klungel O. H. Erkens J. A. Rutten G. E. Leufkens H. G. (2004). Initiation of Glucose-Lowering Therapy in Type 2 Diabetes Mellitus Patients in General Practice. Diabet Med. 21 (8), 896–900. 10.1111/j.1464-5491.2004.01273.x 15270794

[B46] Stone M. A. Charpentier G. Doggen K. Kuss O. Lindblad U. Kellner C. (2013). Quality of Care of People with Type 2 Diabetes in Eight European Countries: Findings from the Guideline Adherence to Enhance Care (GUIDANCE) Study. Diabetes Care 36 (9), 2628–2638. 10.2337/dc12-1759 23628621 PMC3747883

[B47] Tran A. T. Berg T. J. Mdala I. Gjelsvik B. Cooper J. G. Sandberg S. (2021). Factors Associated with Potential over- and Undertreatment of Hyperglycaemia and Annual Measurement of HbA1c in Type 2 Diabetes in Norwegian General Practice. Diabet Med. 38 (8), e14500. 10.1111/dme.14500 33354827 PMC8359382

[B48] van Hateren K. J. Drion I. Kleefstra N. Groenier K. H. Houweling S. T. van der Meer K. (2012). A Prospective Observational Study of Quality of Diabetes Care in a Shared Care Setting: Trends and Age Differences (ZODIAC-19). BMJ Open 2 (4). 10.1136/bmjopen-2012-001387 PMC3432849 22936821

[B50] Verenso (2011). Verantwoorde diabeteszorg bij kwetsbare ouderen thuis en in verzorgings of Verpleeghuizen. Deel 1. [Multidisciplinary guideline diabetes. Responsible diabetes care in vulnerable elderly at home and in residential care or nursing homes. Part 1]. Utrecht: Verenso. Available at: https://www.verenso.nl .

[B51] Vogel B. Acevedo M. Appelman Y. Bairey Merz C. N. Chieffo A. Figtree G. A. (2021). The Lancet Women and Cardiovascular Disease Commission: Reducing the Global burden by 2030. The Lancet 397 (10292), 2385–2438. 10.1016/s0140-6736(21)00684-x 34010613

[B52] Warnes C. A. Williams R. G. Bashore T. M. Child J. S. Connolly H. M. Dearani J. A. (2008). ACC/AHA 2008 Guidelines for the Management of Adults with Congenital Heart Disease: a Report of the American College of Cardiology/American Heart Association Task Force on Practice Guidelines (Writing Committee to Develop Guidelines on the Management of Adults with Congenital Heart Disease). Developed in Collaboration with the American Society of Echocardiography, Heart Rhythm Society, International Society for Adult Congenital Heart Disease, Society for Cardiovascular Angiography and Interventions, and Society of Thoracic Surgeons. J. Am. Coll. Cardiol. 52 (23), e143–e263. 10.1016/j.jacc.2008.10.001 19038677

[B53] Whelton P. K. Carey R. M. Aronow W. S. Casey D. E. Jr. Collins K. J. Dennison Himmelfarb C. (2017). 2017 ACC/AHA/AAPA/ABC/ACPM/AGS/APhA/ASH/ASPC/NMA/PCNA Guideline for the Prevention, Detection, Evaluation, and Management of High Blood Pressure in Adults: Executive Summary: A Report of the American College of Cardiology/American Heart Association Task Force on Clinical Practice Guidelines/ACPM/AGS/APhA/ASH/ASPC/NMA/PCNA Guideline for the Prevention, Detection, Evaluation, and Management of High Blood Pressure in Adults: A Report of the American College of Cardiology/American Heart Association Task Force on Clinical Practice Guidelines. J. Am. Coll. Cardiol. 71 (19), 2199–2269. 10.1016/j.jacc.2017.11.005 29146533

[B54] Williams B. Mancia G. Spiering W. Rosei E. A. Azizi M. Burnier M. (2018). 2018 ESC/ESH Guidelines for the Management of Arterial Hypertension. The Task Force for the Management of Arterial Hypertension of the European Society of Cardiology (ESC) and the European Society of Hypertension (ESH). G Ital. Cardiol. (Rome) 19 (33), 3S–73S. 10.1714/3026.30245 30520455

[B55] Wright A. K. Welsh P. Gill J. M. R. Kontopantelis E. Emsley R. Buchan I. (2020). Age-, Sex- and Ethnicity-Related Differences in Body Weight, Blood Pressure, HbA1c and Lipid Levels at the Diagnosis of Type 2 Diabetes Relative to People without Diabetes. Diabetologia 63 (8), 1542–1553. 10.1007/s00125-020-05169-6 32435821 PMC7351865

