# Supplementary material for: Less Timely Initiation of Glucose-Lowering Medication Among Younger and Male Patients With Diabetes and Similar Initiation of Blood Pressure-Lowering Medication Across Age and Sex: Trends Between 2015 and 2020
Source: Front Pharmacol. 2022 May 12;13:883103. doi: 10.3389/fphar.2022.883103 (PMC9133603; doi:10.3389/fphar.2022.883103)
Supplement: Supplementary file 1 [file DataSheet1.docx]

**Supplementary material 1 – HbA1c analyses**


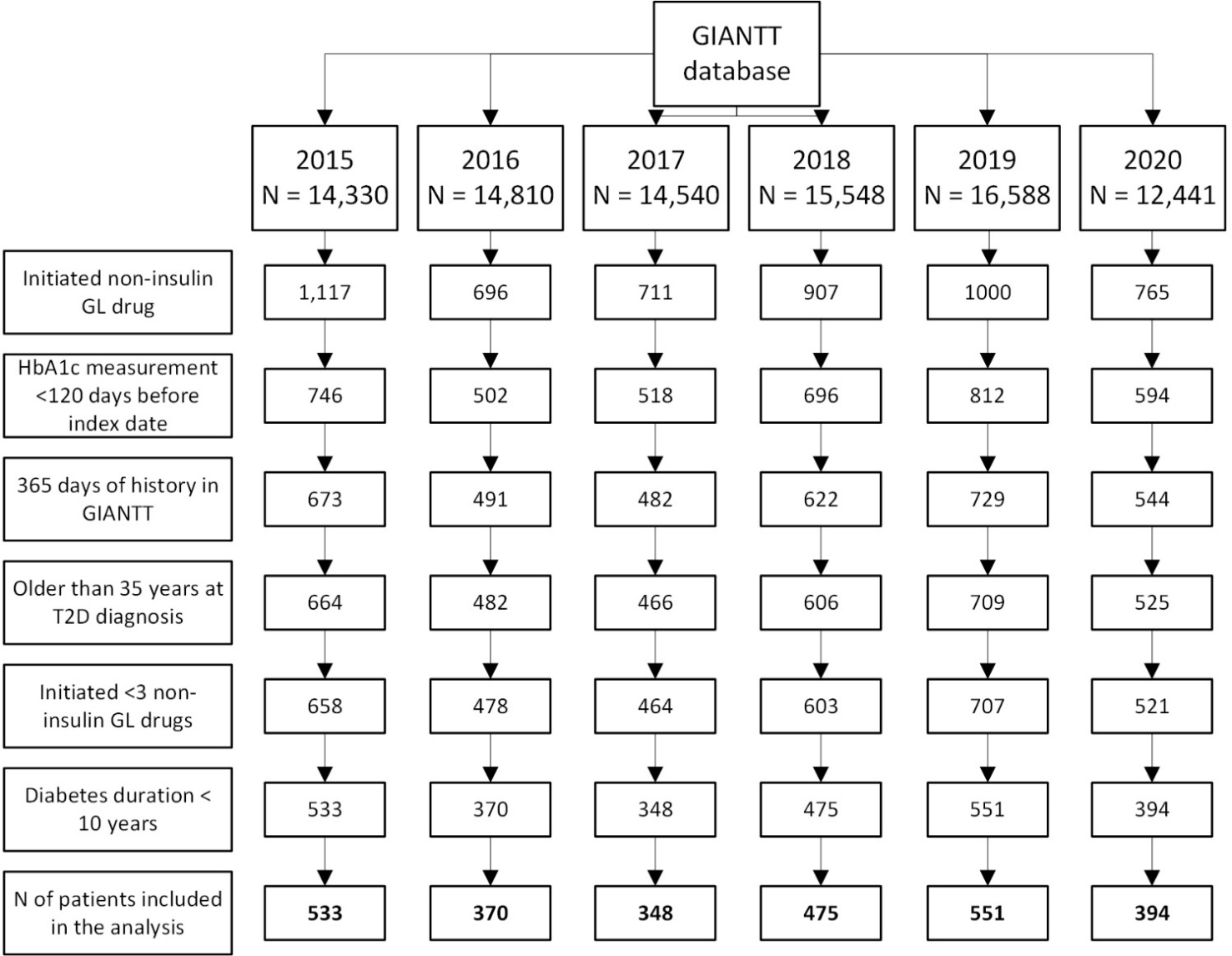


**Supplementary figure 1**: Number of patients per calendar year in the glycosylated hemoglobin A1c (HbA1c) threshold analyses based on the inclusion and exclusion criteria; GIANTT: Groningen Initiative to ANalyze Type 2 diabetes Treatment; GL: glucose-lowering; T2D: type 2 diabetes

**Supplementary table 1**: Characteristics of patients included in the glycosylated hemoglobin A1c (HbA1c) analyses over the years

|  | | | | **2015** | **2016** | **2017** | **2018** | **2019** | **2020** |
| --- | --- | --- | --- | --- | --- | --- | --- | --- | --- |
| **Number of patients** | | | | 533 | 370 | 348 | 475 | 551 | 394 |
| **Females; N (%)** | | | | 242 (45) | 168 (45) | 155 (45) | 227 (48) | 242 (44) | 171 (43) |
| **Age in years; N (%)** | | <60 | | 173 (32) | 115 (31) | 119 (34) | 156 (33) | 192 (35) | 139 (35) |
| 60–69 | | | | 157 (29) | 108 (29) | 114 (33) | 142 (30) | 158 (29) | 113 (29) |
| 70–79 | | | | 124 (23) | 93 (25) | 77 (22) | 125 (26) | 148 (27) | 102 (26) |
| ≥80 | | | | 79 (15) | 54 (15) | 38 (11) | 52 (11) | 53 (10) | 40 (10) |
| **Glycated hemoglobin A1c at initiation in %; mean ± SD** | | | | 7.4 ± 1.4 | 7.4 ± 1.1 | 7.5 ± 1.4 | 7.9 ± 1.5 | 7.7 ± 1.5 | 8.0 ± 1.6 |
| **Fasting glucose; mean ± SD** | | | | 8.7 ± 2.7 | 8.8 ± 2.3 | 9.0 ± 2.7 | 9.3 ± 3.1 | 9.4 ± 3.4 | 9.4 ± 3.2 |
| **Diabetes duration; N (%)** | | 0–1 year | | 241 (45) | 77 (21) | 73 (21) | 192 (40) | 217 (39) | 167 (42) |
| 2–3 years | | | | 87 (16) | 93 (25) | 73 (21) | 70 (15) | 98 (18) | 59 (15) |
| 4–5 years | | | | 81 (15) | 93 (25) | 74 (21) | 94 (20) | 82 (15) | 58 (15) |
| 6–7 years | | | | 58 (11) | 63 (17) | 78 (22) | 82 (17) | 78 (14) | 59 (15) |
| 8–9 years | | | | 66 (12) | 44 (12) | 50 (14) | 37 (8) | 76 (14) | 51 (13) |
| **Systolic blood pressure ≥140 mmHg; N (%)** | | | | 217 (41) | 144 (39) | 137 (39) | 176 (37) | 220 (40) | 161 (41) |
| **Body mass index in kg/m^2^; N (%)** | | <25 | | 66 (12) | 56 (15) | 46 (13) | 58 (12) | 73 (13) | 46 (12) |
| 25–29.9 | | | | 170 (32) | 135 (36) | 134 (39) | 177 (37) | 184 (33) | 148 (38) |
| ≥30 | | | | 274 (51) | 173 (47) | 162 (47) | 230 (48) | 277 (50) | 193 (49) |
| **Dyslipidemia; N (%)** | | | | 284 (53) | 195 (53) | 189 (54) | 254 (53) | 313 (57) | 219 (56) |
| **Estimated glomerular filtration rate ≤60** **mL/min/1.73m^2^; N (%)** | | | | 95 (18) | 76 (21) | 55 (16) | 74 (16) | 85 (15) | 76 (19) |
| **Albuminuria; N (%)** | | | | 1 (0) | 5 (1) | 5 (1) | 9 (2) | 9 (2) | 4 (1) |
| **N of chronic medication at initiation; mean ± SD** | | | | 4.3 ± 3.2 | 4.1 ± 3.0 | 4.3 ± 3.4 | 4.0 ± 3.1 | 4.1 ± 3.0 | 4.0 ± 3.2 |
| **Blood pressure-lowering medication at initiation; N (%)** | | | No medication | 175 (33) | 134 (36) | 124 (36) | 205 (43) | 217 (39) | 170 (43) |
| 1 medication class | | | | 121 (23) | 79 (21) | 90 (26) | 101 (21) | 126 (23) | 82 (21) |
| 2 medication classes | | | | 125 (23) | 83 (22) | 62 (18) | 101 (21) | 120 (22) | 68 (17) |
| 3 or more medication classes | | | | 112 (21) | 74 (20) | 72 (21) | 68 (14) | 88 (16) | 74 (19) |
| **Treated with a lipid-lowering medication; N (%)** | | | | 296 (56) | 206 (56) | 188 (54) | 237 (50) | 273 (50) | 173 (44) |
| **Initiated medication; N (%)** | Metformin | | | 441 (83) | 321 (87) | 288 (83) | 422 (89) | 497 (90) | 359 (91) |
| Sulfonylurea | | | | 42 (8) | 25 (7) | 30 (9) | 26 (6) | 33 (6) | 22 (6) |
| α-glucosidase inhibitors | | | | 1 (0) | - | - | - | - | - |
| DDP-4 inhibitor | | | | - | 1 (0) | - | 2 (0) | 1 (0) | 1 (0) |
| GLP-1 inhibitor | | | | - | - | - | - | - | 2 (1) |
| SGLT2 inhibitor | | | | - | - | - | - | - | 1 (0) |
| Metformin + another medication | | | | 49 (9) | 22 (5) | 29 (8) | 24 (5) | 20 (4) | 7 (2) |
| Sulfonylurea + another medication | | | | - | 1 (0) | 1 (0) | 1 (0) | - | 2 (1) |

| **Supplementary table 2**: Characteristics of patients included in the glycosylated hemoglobin A1c (HbA1c) analyses per age group | | | | | | |
| --- | --- | --- | --- | --- | --- | --- |
|  | | | **<60 years** | **60-69 years** | **70-79 years** | **≥80 years** |
| **Females; N (%)** | | | 391 (44) | 324 (41) | 310 (46) | 180 (57) |
| **Glycated hemoglobin A1c at initiation in %; mean ± SD** | | | 7.9 ± 1.6 | 7.5 ± 1.5 | 7.4 ± 1.2 | 7.7 ± 1.3 |
| **Fasting glucose; mean ± SD** | | | 9.7 ± 3.4 | 8.9 ± 2.8 | 8.7 ± 2.3 | 9.0 ± 3.4 |
| **Diabetes duration; N (%)** | 0 – 1 years | | 408 (46) | 276 (35) | 196 (29) | 87 (28) |
| 2 – 3 years | | | 160 (18) | 156 (20) | 109 (16) | 55 (17) |
| 4 – 5 years | | | 149 (17) | 146 (18) | 127 (19) | 60 (19) |
| 6 – 7 years | | | 114 (13) | 116 (15) | 131 (20) | 57 (18) |
| 8 – 9 years | | | 63 (7) | 98 (12) | 106 (16) | 57 (18) |
| **Systolic blood pressure ≥140 mmHg; N (%)** | | | 277 (31) | 322 (41) | 302 (45) | 154 (49) |
| **Body mass index in kg/m^2^; N (%)** | < 24.9 | | 69 (8) | 101 (13) | 101 (13) | 74 (23) |
| 25 – 29.9 | | | 248 (28) | 296 (37) | 296 (37) | 145 (46) |
| ≥ 30 | | | 551 (62) | 380 (48) | 380 (48) | 82 (26) |
| **Dyslipidemia; N (%)** | | | 528 (59) | 432 (55) | 337 (50) | 157 (50) |
| **Estimated glomerular filtration rate ≤60 ml/min/1.73m2; N (%)** | | | 25 (3) | 93 (12) | 197 (30) | 146 (46) |
| **Albuminuria (%)** | | | 8 (1) | 13 (2) | 6 (1) | 6 (2) |
| **Number of chronic medications at initiation; mean ± SD** | | | 3.4 ± 2.9 | 3.8 ± 2.8 | 5.1 ± 3.3 | 5.2 ± 3.6 |
| **Blood pressure-lowering medication at initiation; N (%)** | | No medication | 469 (52) | 307 (39) | 160 (24) | 89 (28) |
| 1 medication class | | | 184 (21) | 199 (25) | 142 (21) | 74 (23) |
| 2 medication classes | | | 143 (16) | 154 (19) | 199 (30) | 63 (20) |
| 3 or more medication classes | | | 98 (11) | 132 (17) | 168 (25) | 90 (28) |
| **Treated with a lipid-lowering medication; N (%)** | | | 415 (46) | 434 (55) | 384 (57) | 140 (44) |
| **Initiated medication; N (%)** | Metformin | | 797 (89) | 705 (89) | 587 (88) | 239 (76) |
| Sulphonyl urea | | | 43 (5) | 48 (6) | 38 (6) | 49 (16) |
| α-glucosidase inhibitors | | | - | - | - | 1 (0) |
| Dipeptidyl Peptidase 4 (DDP-4) inhibitor | | | 2 (0) | 1 (0) | 1 (0) | 1 (0) |
| Glucagon-like peptide-1 (GLP-1) agonist | | | 2 (0) | - | - | - |
| Sodium-glucose transport protein 2 (SGLT2) inhibitor | | | - | 1 (0) | - | - |
| Metformin + another medication | | | 48 (5) | 36 (4) | 42 (6) | 25 (8) |
| Sulphonyl urea + another medication | | | 2 (0) | 1 (0) | 1 (0) | 1 (0) |

**Supplementary table 3**: Characteristics of patients included in the glycosylated hemoglobin A1c (HbA1c) analyses per sex group

|  | | | **Males** | **Females** |
| --- | --- | --- | --- | --- |
| **Age group** < 60 | | | 503 (34) | 391 (32) |
| 60-69 | | | 468 (32) | 324 (27) |
| 70-79 | | | 359 (24) | 310 (26) |
| ≥80 | | | 136 (9) | 180 (15) |
| **Glycated hemoglobin A1c at initiation in %; mean ± SD** | | | 7.8 ± 1.5 | 7.5 ± 1.4 |
| **Fasting glucose; mean ± SD** | | | 9.3 ± 3.1 | 8.9 ± 2.8 |
| **Diabetes duration; N (%)** | 0 – 1 years | | 547 (37) | 420 (25) |
| 2 – 3 years | | | 287 (20) | 193 (16) |
| 4 – 5 years | | | 248 (17) | 234 (19) |
| 6 – 7 years | | | 218 (15) | 200 (17) |
| 8 – 9 years | | | 166 (11) | 158 (13) |
| **Systolic blood pressure ≥140 mmHg; N (%)** | | | 568 (39) | 487 (40) |
| **Body mass index in kg/m^2^; N (%)** | < 24.9 | | 175 (12) | 170 (14) |
| 25 – 29.9 | | | 586 (40) | 362 (30) |
| ≥ 30 | | | 678 (46) | 631 (52) |
| **Dyslipidemia; N (%)** | | | 765 (52) | 689 (57) |
| **Estimated glomerular filtration rate ≤ 60 ml/min/1.73m2; N (%)** | | | 222 (15) | 239 (20) |
| **Albuminuria (%)** | | | 26 (2) | 7 (1) |
| **Number of chronic medications at initiation; mean ± SD** | | | 3.8 ± 2.9 | 4.6 ± 3.3 |
| **Blood pressure-lowering treatment at initiation; N (%)** | | No treatment | 590 (40) | 435 (36) |
| 1 medication class | | | 321 (22) | 278 (23) |
| 2 medication classes | | | 288 (20) | 271 (22) |
| 3 or more medication classes | | | 267 (18) | 221 (18) |
| **Treated with a lipid-lowering drug; N (%)** | | | 775 (53) | 589 (50) |
| **Initiated medication; N (%)** | Metformin | | 1,274 (87) | 1,054 (87) |
| Sulphonyl urea | | | 94 (6) | 84 (7) |
| α-glucosidase inhibitors | | | - | 1 (0) |
| Dipeptidyl Peptidase 4 (DDP-4) inhibitor | | | 2 (0) | 3 (0) |
| Glucagon-like peptide-1 (GLP-1) agonist | | | 1 (0) | 1 (0) |
| Sodium-glucose transport protein 2 (SGLT2) inhibitor | | | 1 (0) | - |
| Metformin + another medication | | | 92 (6) | 59 (5) |
| Sulphonyl urea + another medication | | | 2 (0) | 3 (0) |

**Supplementary material 2 – SBP analyses**


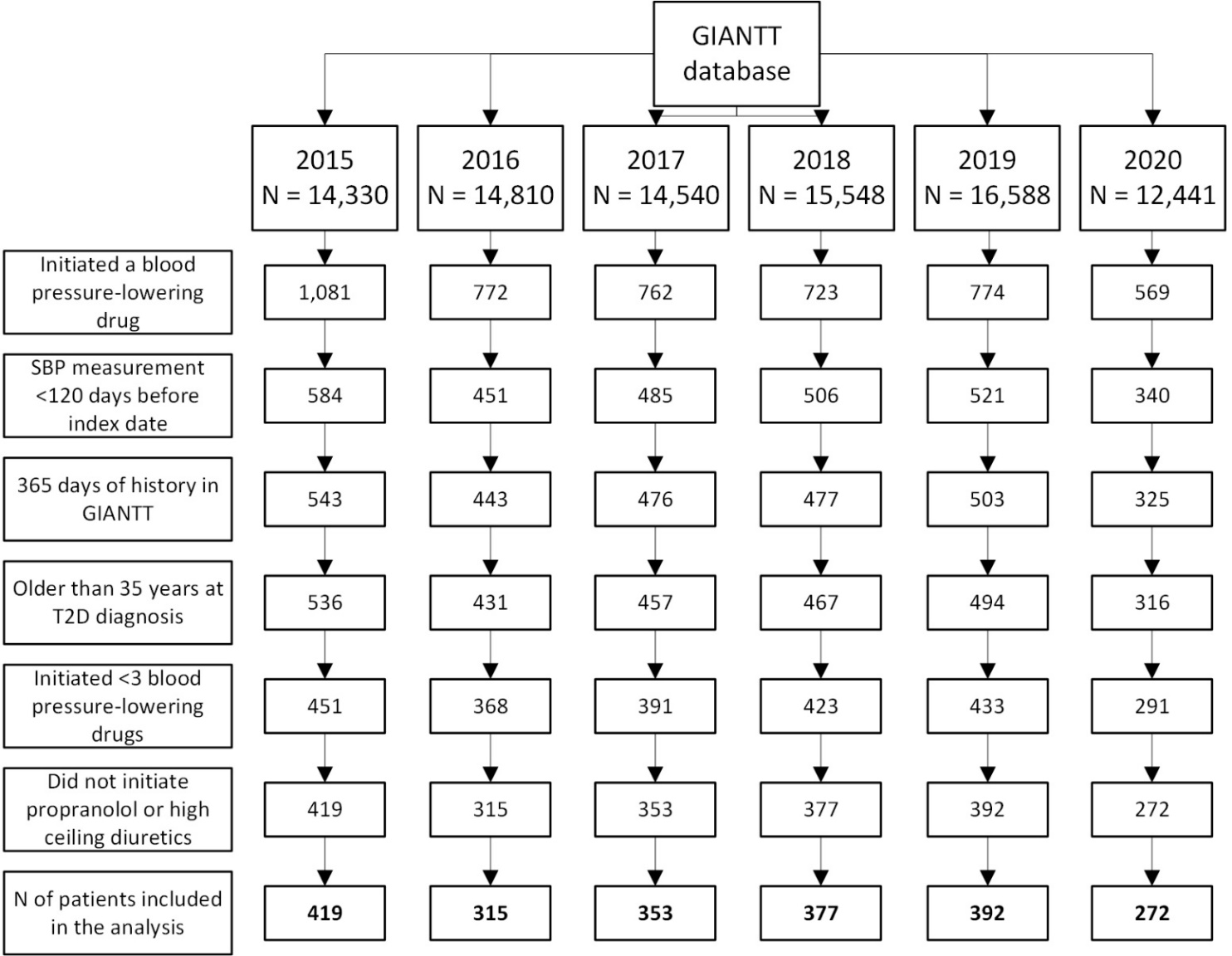


**Supplementary figure 2**: Numbers of patients per calendar year in the systolic blood pressure (SBP) analyses based on the inclusion and exclusion criteria; GIANTT: Groningen Initiative to ANalyze Type 2 diabetes Treatment; T2D: type 2 diabetes

**Supplementary table 4**: Characteristics of patients included in the systolic blood pressure (SBP) analyses over the years

|  | | | | | **2015** | **2016** | **2017** | **2018** | **2019** | **2020** |
| --- | --- | --- | --- | --- | --- | --- | --- | --- | --- | --- |
| **Number of patients** | | | | | 419 | 315 | 353 | 377 | 392 | 272 |
| **Females; N (%)** | | | | | 223 (53) | 152 (48) | 154 (44) | 170 (45) | 188 (48) | 124 (46) |
| **Age in years; N (%)** | | <60 | | | 99 (24) | 81 (26) | 101 (29) | 112 (30) | 94 (24) | 72 (26) |
| 60–69 | | | | | 128 (31) | 96 (30) | 100 (28) | 111 (29) | 124 (32) | 91 (33) |
| 70–79 | | | | | 105 (25) | 80 (25) | 102 (29) | 100 (27) | 114 (29) | 65 (24) |
| ≥80 | | | | | 87 (21) | 58 (18) | 50 (14) | 54 (14) | 60 (15) | 44 (16) |
| **Systolic BP at initiation in mmHg; mean ± SD** | | | | | 145 ± 21 | 147 ± 20 | 148 ± 21 | 146 ± 21 | 145 ± 20 | 149 ± 21 |
| **Diastolic BP at initiation in mmHg; mean ± SD** | | | | | 80 ± 13 | 81 ± 13 | 83 ± 13 | 81 ± 12 | 81 ± 12 | 83 ± 12 |
| **Diabetes duration; N (%)** | | 0–1 year | | | 69 (16) | 35 (11) | 17 (5) | 57 (15) | 55 (14) | 40 (15) |
| 2–3 years | | | | | 57 (14) | 58 (18) | 57 (16) | 53 (14) | 56 (14) | 35 (13) |
| 4–5 years | | | | | 57 (14) | 41 (13) | 55 (16) | 48 (13) | 42 (11) | 33 (12) |
| 6–7 years | | | | | 62 (15) | 47 (15) | 58 (16) | 54 (14) | 52 (13) | 34 (13) |
| 8–9 years | | | | | 61 (15) | 32 (10) | 44 (12) | 36 (10) | 51 (13) | 26 (10) |
| ≥10 years | | | | | 113 (27) | 102 (32) | 122 (35) | 129 (34) | 136 (35) | 104 (38) |
| **Glycated hemoglobin A1c < 7%; N (%)** | | | | | 207 (49) | 162 (51) | 187 (53) | 183 (49) | 209 (53) | 124 (46) |
| **Body mass index in kg/m^2^; N (%)** | | <25 | | | 73 (17) | 50 (16) | 61 (17) | 81 (21) | 76 (19) | 51 (19) |
| 25–29.9 | | | | | 168 (40) | 123 (39) | 154 (44) | 129 (34) | 152 (39) | 97 (36) |
| ≥30 | | | | | 169 (40) | 136 (43) | 130 (37) | 157 (42) | 157 (40) | 117 (43) |
| **Dyslipidemia; N (%)** | | | | | 183 (54) | 142 (58) | 162 (58) | 164 (50) | 187 (56) | 116 (51) |
| **Estimated glomerular filtration rate ≤60** **mL/min/1.73m^2^; N (%)** | | | | | 76 (18) | 69 (22) | 41 (12) | 69 (18) | 70 (18) | 43 (16) |
| **Albuminuria; N (%)** | | | | | 11 (3) | 14 (4) | 10 (3) | 14 (4) | 12 (3) | 7 (3) |
| **Smoking; N (%)** | | | | | 80 (19) | 53 (17) | 50 (14) | 79 (21) | 66 (17) | 43 (16) |
| **History of cardiovascular disease; N (%)** | | | Myocardial disease | | 60 (14) | 34 (11) | 30 (9) | 32 (8) | 33 (8) | 24 (9) |
| Heart failure | | | | | 33 (8) | 10 (3) | 17 (5) | 11 (3) | 8 (2) | 5 (2) |
| Stroke | | | | | 31 (7) | 18 (6) | 23 (7) | 15 (4) | 20 (5) | 7 (3) |
| **Number of chronic medications at initiation; mean ± SD** | | | | | 4.0 ± 2.7 | 3.8 ± 2.9 | 3.7± 2.7 | 3.7 ± 2.8 | 3.7 ± 2.8 | 3.8 ± 2.8 |
| **Glucose-lowering medication at initiation; N (%)** | | | | No medication | 147 (35) | 100 (32) | 118 (33) | 141 (37) | 165 (42) | 89 (33) |
| 1 oral | | | | | 153 (37) | 115 (37) | 125 (35) | 131 (35) | 129 (33) | 83 (31) |
| 2 orals | | | | | 64 (15) | 65 (21) | 52 (15) | 41 (11) | 46 (12) | 50 (18) |
| 3 orals or more and/or insulin | | | | | 55 (13) | 35 (11) | 58 (16) | 64 (17) | 52 (13) | 50 (18) |
| **Treated with lipid lowering medication; N (%)** | | | | | 215 (51) | 176 (56) | 184 (52) | 175 (46) | 195 (50) | 128 (47) |
| **Initiated medication class; N (%)** | Renin-angiotensin-aldosterone system inhibitor | | | | 148 (35) | 136 (43) | 156 (44) | 152 (40) | 155 (40) | 123 (45) |
|  | Combination of antihypertensives | | | | 107 (26) | 68 (22) | 68 (19) | 73 (19) | 80 (20) | 49 (18) |
| Beta blocker | | | | | 75 (18) | 46 (15) | 47 (13) | 50 (13) | 76 (19) | 51 (19) |
| Diuretic | | | | | 54 (13) | 38 (12) | 50 (14) | 61 (16) | 37 (9) | 20 (7) |
| Calcium channel blocker | | | | | 35 (8) | 27 (9) | 32 (9) | 41 (11) | 44 (11) | 29 (11) |

| **Supplementary table 5**: Characteristics of patients included in the systolic blood pressure (SBP) analyses per age group | | | | | | | | |
| --- | --- | --- | --- | --- | --- | --- | --- | --- |
|  | | | | | **<60 years** | **60-69 years** | **70-79 years** | **≥80 years** |
| **Females; N (%)** | | | | | 233 (42) | 281 (43) | 274 (48) | 223 (63) |
| **Systolic BP at initiation in mmHg; mean ± SD** | | | | | 146 ± 20 | 147 ± 21 | 147 ± 21 | 143 ± 21 |
| **Diastolic BP at initiation in mmHg; mean ± SD** | | | | | 88 ± 12 | 83 ± 12 | 78 ± 11 | 75 ± 12 |
| **Diabetes duration; N (%)** | | 0 – 1 years | | | 117 (21) | 80 (12) | 52 (9) | 24 (7) |
|  | | 2 – 3 years | | | 123 (22) | 94 (14) | 64 (11) | 35 (10) |
|  | | 4 – 5 years | | | 82 (15) | 95 (15) | 65 (11) | 34 (10) |
|  | | 6 – 7 years | | | 85 (15) | 109 (17) | 73 (13) | 40 (11) |
|  | | 8 – 9 years | | | 64 (11) | 74 (11) | 72 (13) | 40 (11) |
|  | | ≥ 10 years | | | 88 (16) | 198 (30) | 240 (42) | 180 (51) |
| **Glycated hemoglobin A1c < 7%; N (%)** | | | | | 264 (47) | 335 (52) | 294 (52) | 179 (51) |
| **Body mass index in kg/m^2^; N (%)** | | | < 24.9 | | 41 (7) | 119 (18) | 120 (21) | 112 (32) |
| 25 – 29.9 | | | | | 164 (29) | 263 (40) | 246 (43) | 150 (42) |
| ≥ 30 | | | | | 339 (60) | 256 (39) | 188 (33) | 83 (24) |
| **Dyslipidemia; N (%)** | | | | | 270 (48) | 306 (47) | 245 (43) | 133 (38) |
| **Estimated glomerular filtration rate ≤60 ml/min/1.73m2; N (%)** | | | | | 17 (3) | 51 (8) | 124 (22) | 176 (50) |
| **Albuminuria (%)** | | | | | 16 (3) | 18 (3) | 16 (3) | 18 (5) |
| **Smoking; N (%)** | | | | | 139 (25) | 129 (20) | 80 (14) | 23 (7) |
| **History of cardiovascular disease; N (%)** | | | Myocardial disease^1^ | | 21 (4) | 61 (9) | 71 (13) | 60 (17) |
| Heart failure^2^ | | | | | 3 (1) | 13 (2) | 24 (4) | 44 (12) |
| Stroke^3^ | | | | | 8 (1) | 25 (4) | 42 (7) | 39 (11) |
| **Number of chronic medications at initiation; mean ± SD** | | | | | 3.8 ± 2.6 | 3.7 ± 2.8 | 3.8 ± 2.8 | 4.4 ± 3.0 |
| **Glucose-lowering medication at initiation; N (%)** | | | | No medication | 183 (33) | 225 (35) | 208 (37) | 144 (41) |
| 1 oral | | | | | 192 (34) | 231 (26) | 201 (36) | 112 (32) |
| 2 orals | | | | | 87 (16) | 112 (17) | 77 (14) | 42 (12) |
| 3 orals or more and/or insulin | | | | | 97 (17) | 82 (13) | 80 (14) | 55 (16) |
| **Treated with lipid-lowering medication; N (%)** | | | | | 289 (52) | 375 (58) | 286 (51) | 123 (35) |
| **Initiated medication class; N (%)** | Renin-angiotensin-aldosterone system inhibitor | | | | 298 (62) | 284 (54) | 197 (45) | 91 (38) |
|  | Combination of antihypertensives | | | | 79 (14) | 121 (19) | 132 (23) | 113 (32) |
|  | Beta blocker | | | | 62 (13) | 90 (17) | 115 (27) | 78 (33) |
| Diuretic | | | | | 75 (16) | 90 (17) | 52 (12) | 43 (18) |
| Calcium channel blocker | | | | | 45 (9) | 65 (12) | 70 (16) | 28 (12) |

**Supplementary table 6**: Characteristics of patients included in the systolic blood pressure (SBP) analyses per sex group

|  | | | | | **Males** | **Females** |
| --- | --- | --- | --- | --- | --- | --- |
| **Age group** <60 | | | | | 326 (29) | 233 (23) |
| 60-69 | | | | | 369 (33) | 281 (28) |
| 70-79 | | | | | 292 (26) | 274 (27) |
| ≥80 | | | | | 130 (12) | 223 (23) |
| **Systolic BP at initiation in mmHg; mean ± SD** | | | | | 147 ± 21 | 146 ± 21 |
| **Diastolic BP at initiation in mmHg; mean ± SD** | | | | | 82 ± 13 | 80 ± 12 |
| **Diabetes duration; N (%)** | | 0 – 1 years | | | 161 (14) | 112 (11) |
|  | | 2 – 3 years | | | 185 (17) | 131 (13) |
|  | | 4 – 5 years | | | 131 (12) | 145 (14) |
|  | | 6 – 7 years | | | 159 (14) | 148 (15) |
|  | | 8 – 9 years | | | 126 (11) | 124 (12) |
|  | | ≥ 10 years | | | 355 (32) | 351 (35) |
| **Glycated hemoglobin A1c < 7%; N (%)** | | | | | 544 (49) | 528 (52) |
| **Body mass index in kg/m^2^; N (%)** | | | < 24.9 | | 193 (17) | 199 (20) |
| 25 – 29.9 | | | | | 487 (44) | 336 (33) |
| ≥ 30 | | | | | 410 (37) | 456 (45) |
| **Dyslipidemia; N (%)** | | | | | 488 (44) | 466 (46) |
| **Estimated glomerular filtration rate ≤60 ml/min/1.73m2; N (%)** | | | | | 170 (15) | 198 (20) |
| **Albuminuria (%)** | | | | | 46 (4) | 22 (2) |
| **Smoking; N (%)** | | | | | 205 (18) | 166 (16) |
| **History of cardiovascular disease; N (%)** | | | Myocardial disease^1^ | | 144 (13) | 69 (7) |
| Heart failure^2^ | | | | | 40 (4) | 44 (4) |
| Stroke^3^ | | | | | 54 (5) | 60 (6) |
| **Number of chronic medications at initiation; mean ± SD** | | | | | 3.5 ± 2.6 | 4.1 ± 2.9 |
| **Glucose-lowering medication at initiation; N (%)** | | | | No medication | 374 (33) | 386 (38) |
| 1 oral | | | | | 396 (35) | 340 (34) |
| 2 orals | | | | | 191 (17) | 127 (13) |
| 3 orals or more and/or insulin | | | | | 156 (14) | 158 (16) |
| **Treated with a lipid-lowering medication; N (%)** | | | | | 602 (54) | 471 (47) |
| **Initiated medication class; N (%)** | Renin-angiotensin-aldosterone system inhibitor | | | | 512 (57) | 358 (46) |
|  | Combination of antihypertensives | | | | 218 (20) | 227 (22) |
|  | Beta blocker | | | | 180 (20) | 165 (21) |
| Diuretic | | | | | 101 (11) | 159 (20) |
| Calcium channel blocker | | | | | 106 (12) | 102 (13) |

**Supplementary material 3 – Sex differences in the years 2007 to 2014**

*Ambroz M, de Vries ST, Hoogenberg K, Denig P*. Trends in HbA1c thresholds for initiation of hypoglycemic agents: Impact of changed recommendations for older and frail patients. Pharmcoepidemiology and Drug Safety. 2021;30(1):37-44.

*Ambroz M, de Vries ST, Sidorenkov G, Hoogenberg K, Denig P*. Changes in blood pressure thresholds for initiating antihypertensive medication in patients with diabetes: a repeated cross-sectional study focusing on the impact of age and frailty. BMJ Open. 2020;10(9):1-8. e037694.


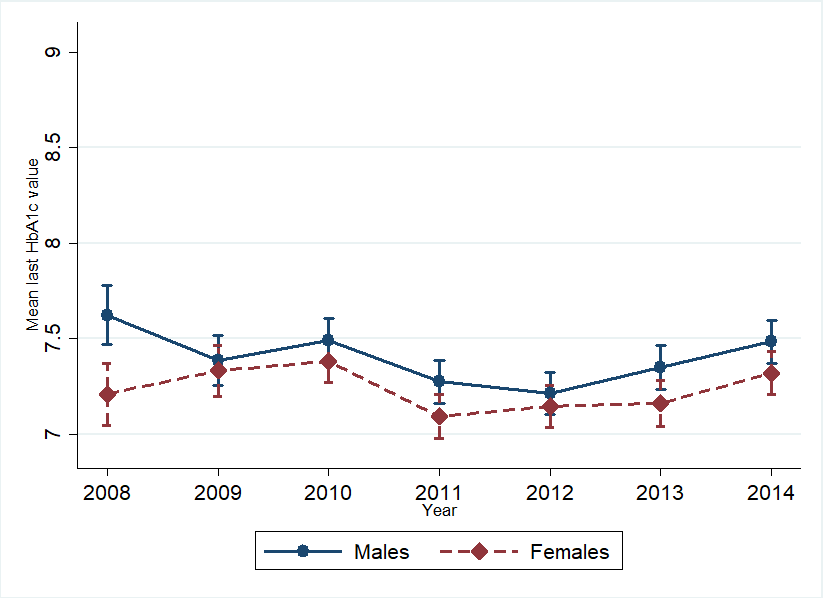

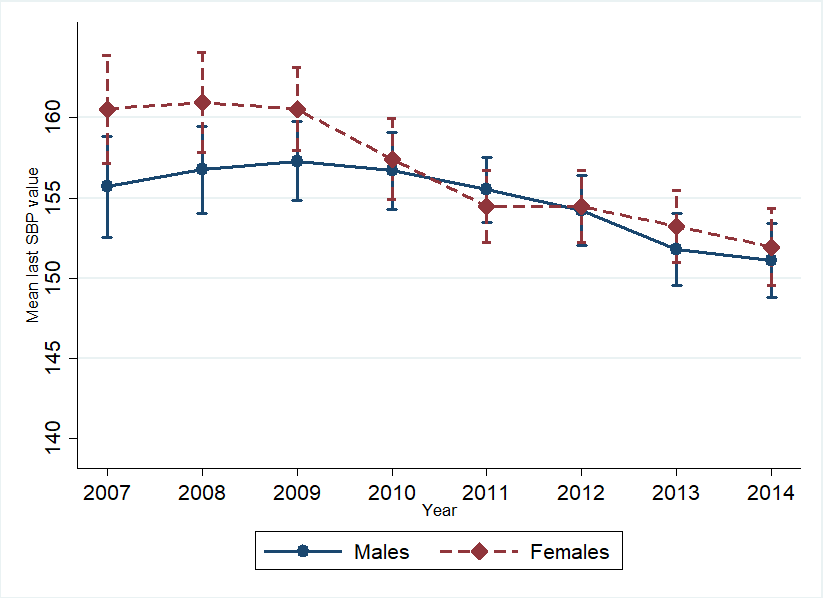


b

a

**Supplementary figure 3:** Mean last (a) glycated hemoglobin A1c (HbA1c) levels before/at initiation of glucose-lowering medication between 2008 and 2014 and (b) systolic blood pressure (SBP) levels before/at initiation of blood pressure lowering medication between 2007 and 2014. Mean levels are adjusted for confounders and presented with 95% confidence intervals.
